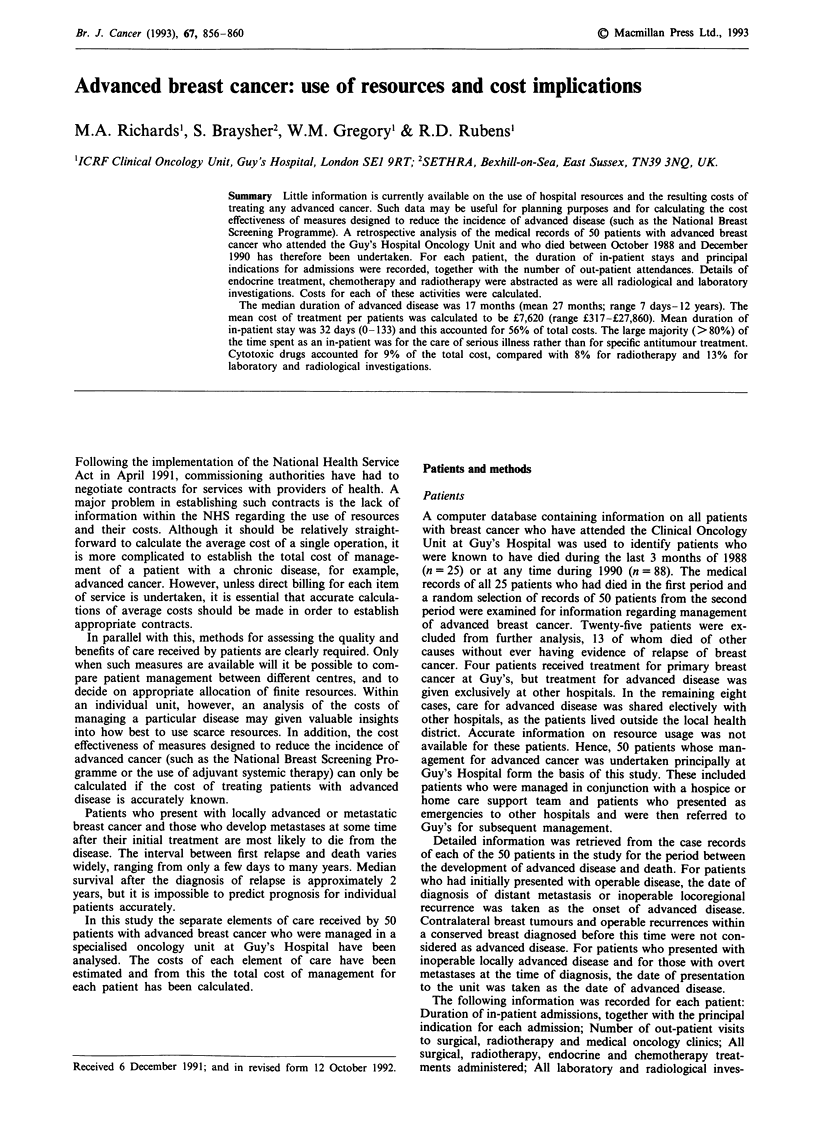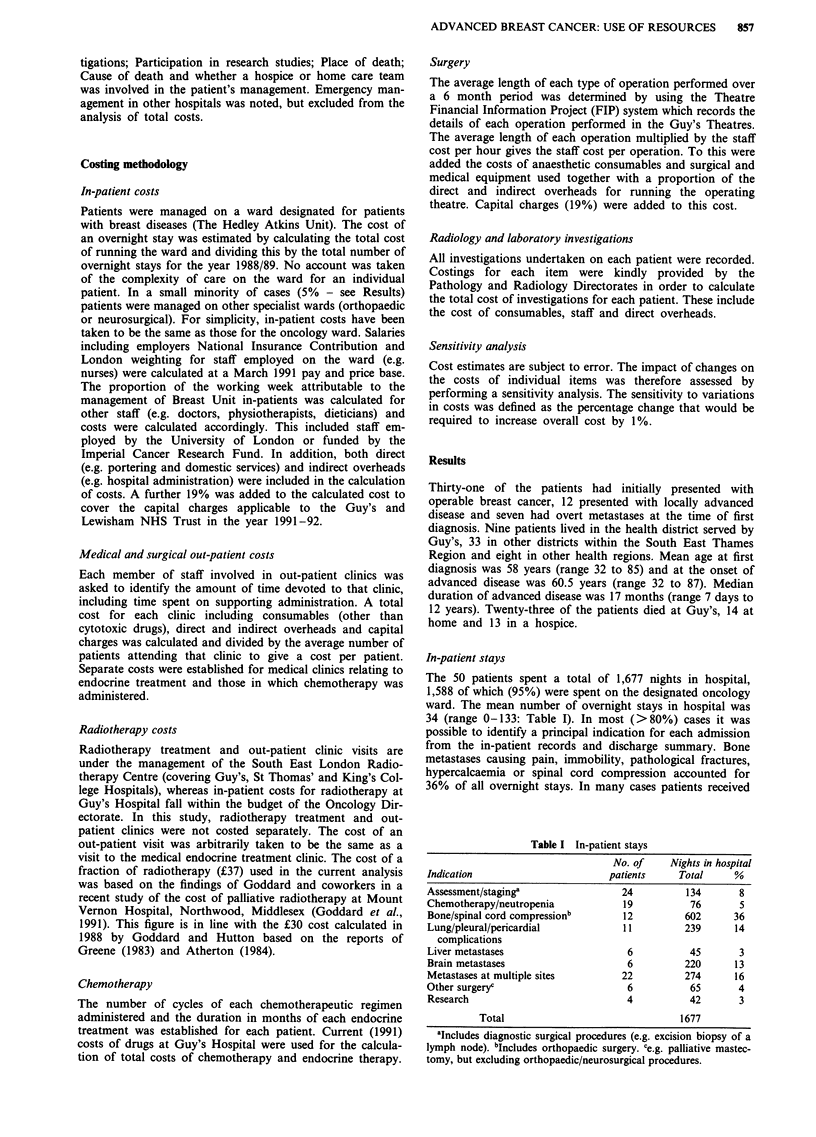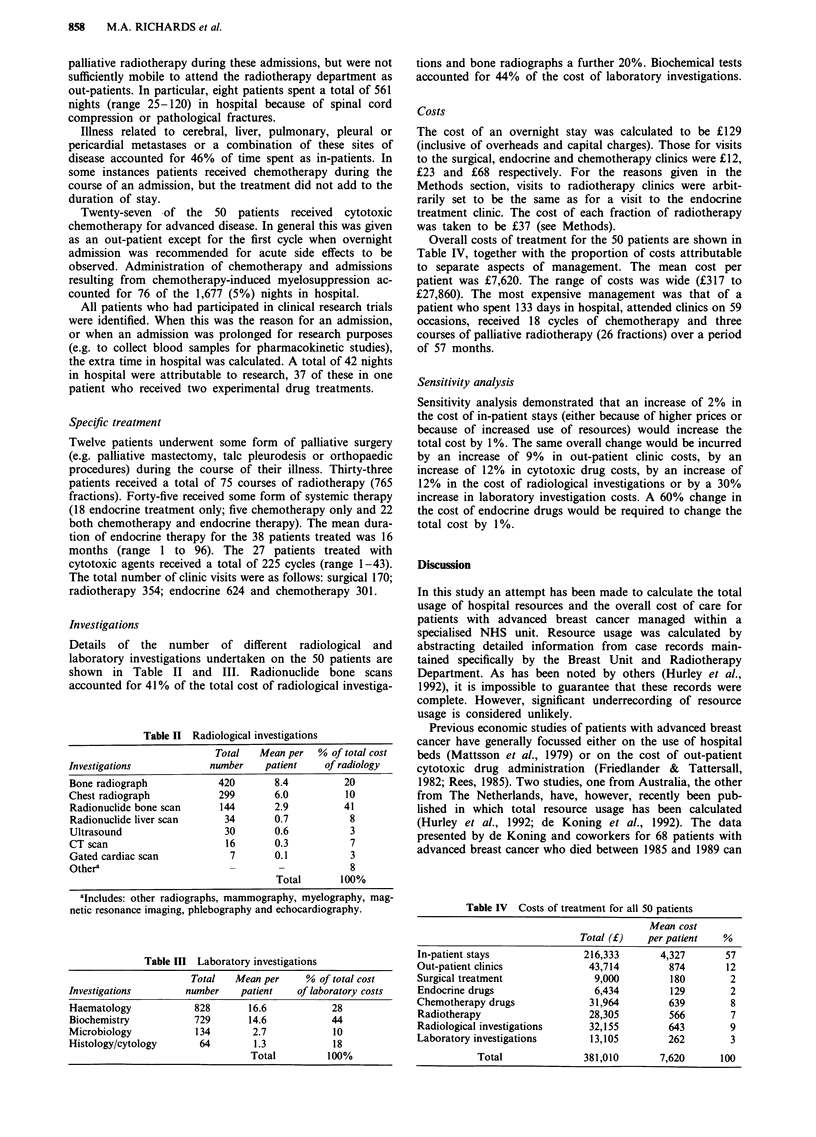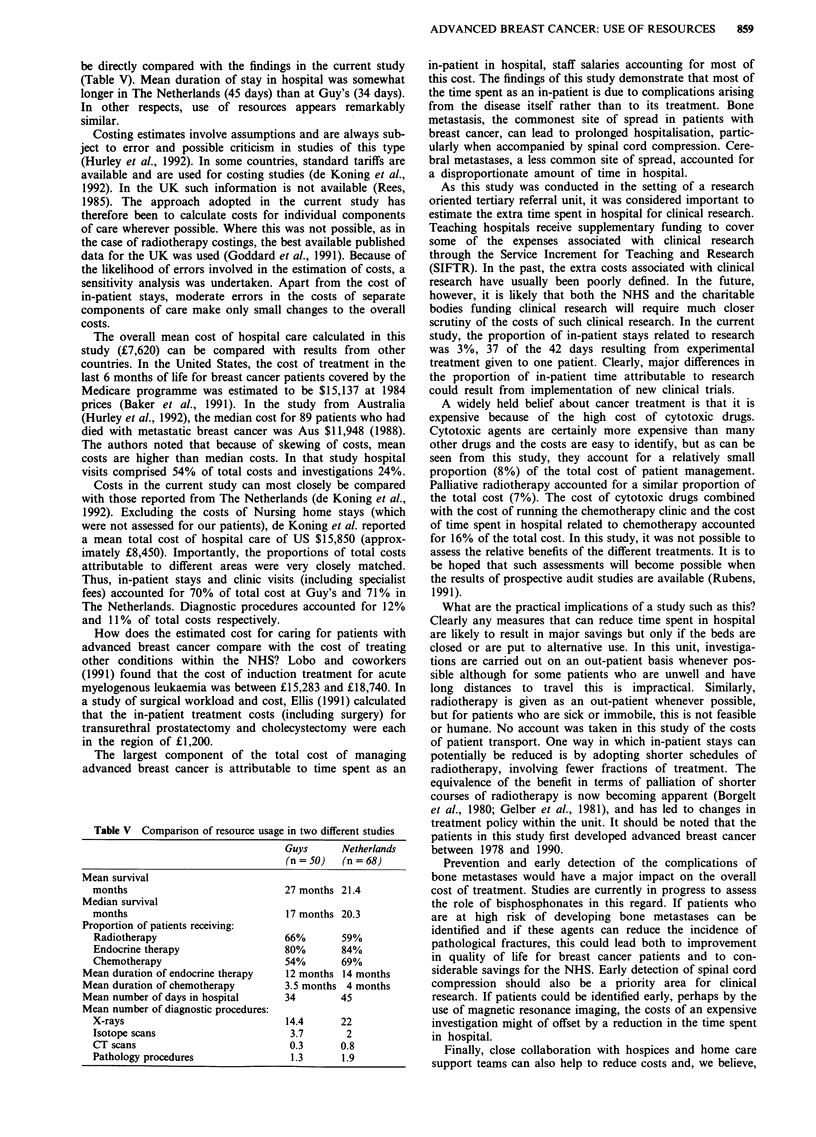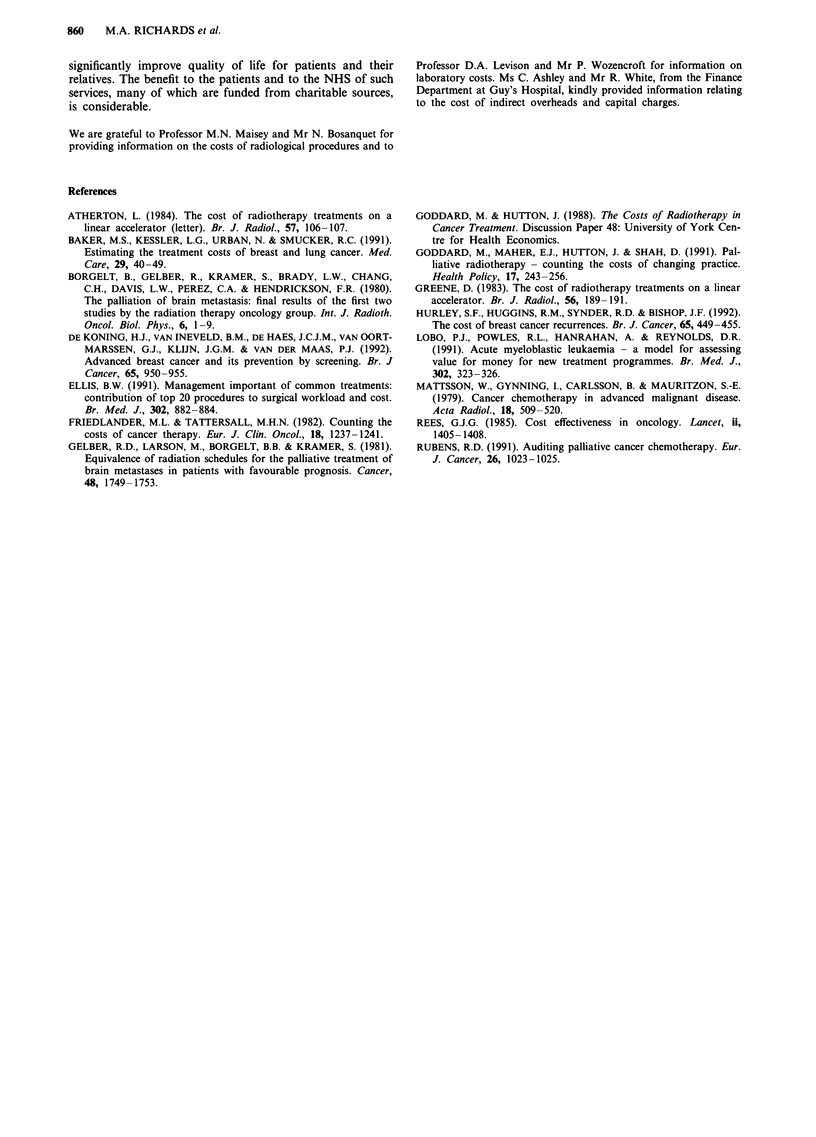# Advanced breast cancer: use of resources and cost implications.

**DOI:** 10.1038/bjc.1993.157

**Published:** 1993-04

**Authors:** M. A. Richards, S. Braysher, W. M. Gregory, R. D. Rubens

**Affiliations:** ICRF Clinical Oncology Unit, Guy's Hospital, London, UK.

## Abstract

Little information is currently available on the use of hospital resources and the resulting costs of treating any advanced cancer. Such data may be useful for planning purposes and for calculating the cost effectiveness of measures designed to reduce the incidence of advanced disease (such as the National Breast Screening Programme). A retrospective analysis of the medical records of 50 patients with advanced breast cancer who attended the Guy's Hospital Oncology Unit and who died between October 1988 and December 1990 has therefore been undertaken. For each patient, the duration of in-patient stays and principal indications for admissions were recorded, together with the number of out-patient attendances. Details of endocrine treatment, chemotherapy and radiotherapy were abstracted as were all radiological and laboratory investigations. Costs for each of these activities were calculated. The median duration of advanced disease was 17 months (mean 27 months; range 7 days-12 years). The mean cost of treatment per patients was calculated to be 7,620 pounds (range 317 pounds-27,860 pounds). Mean duration of in-patient stay was 32 days (0-133) and this accounted for 56% of total costs. The large majority (> 80%) of the time spent as an in-patient was for the care of serious illness rather than for specific antitumour treatment. Cytotoxic drugs accounted for 9% of the total cost, compared with 8% for radiotherapy and 13% for laboratory and radiological investigations.


*Br. J. Cancer* (1993), **67**, 856–860 © Macmillan Press Ltd., 1993

### Advanced breast cancer: use of resources and cost implications

M.A. Richards[1], S. Braysher[2], W.M. Gregory[1] & R.D. Rubens[1]

[1]*ICRF Clinical Oncology Unit, Guy's Hospital, London SE1 9RT;* [2]*SETHRA, Bexhill-on-Sea, East Sussex, TN39 3NQ, UK.*

**Summary** Little information is currently available on the use of hospital resources and the resulting costs of treating any advanced cancer. Such data may be useful for planning purposes and for calculating the cost effectiveness of measures designed to reduce the incidence of advanced disease (such as the National Breast Screening Programme). A retrospective analysis of the medical records of 50 patients with advanced breast cancer who attended the Guy's Hospital Oncology Unit and who died between October 1988 and December 1990 has therefore been undertaken. For each patient, the duration of in-patient stays and principal indications for admissions were recorded, together with the number of out-patient attendances. Details of endocrine treatment, chemotherapy and radiotherapy were abstracted as were all radiological and laboratory investigations. Costs for each of these activities were calculated.

The median duration of advanced disease was 17 months (mean 27 months; range 7 days–12 years). The mean cost of treatment per patients was calculated to be £7,620 (range £317–£27,860). Mean duration of in-patient stay was 32 days (0–133) and this accounted for 56% of total costs. The large majority (>80%) of the time spent as an in-patient was for the care of serious illness rather than for specific antitumour treatment. Cytotoxic drugs accounted for 9% of the total cost, compared with 8% for radiotherapy and 13% for laboratory and radiological investigations.

Following the implementation of the National Health Service Act in April 1991, commissioning authorities have had to negotiate contracts for services with providers of health. A major problem in establishing such contracts is the lack of information within the NHS regarding the use of resources and their costs. Although it should be relatively straightforward to calculate the average cost of a single operation, it is more complicated to establish the total cost of management of a patient with a chronic disease, for example, advanced cancer. However, unless direct billing for each item of service is undertaken, it is essential that accurate calculations of average costs should be made in order to establish appropriate contracts.

In parallel with this, methods for assessing the quality and benefits of care received by patients are clearly required. Only when such measures are available will it be possible to compare patient management between different centres, and to decide on appropriate allocation of finite resources. Within an individual unit, however, an analysis of the costs of managing a particular disease may given valuable insights into how best to use scarce resources. In addition, the cost effectiveness of measures designed to reduce the incidence of advanced cancer (such as the National Breast Screening Programme or the use of adjuvant systemic therapy) can only be calculated if the cost of treating patients with advanced disease is accurately known.

Patients who present with locally advanced or metastatic breast cancer and those who develop metastases at some time after their initial treatment are most likely to die from the disease. The interval between first relapse and death varies widely, ranging from only a few days to many years. Median survival after the diagnosis of relapse is approximately 2 years, but it is impossible to predict prognosis for individual patients accurately.

In this study the separate elements of care received by 50 patients with advanced breast cancer who were managed in a specialised oncology unit at Guy's Hospital have been analysed. The costs of each element of care have been estimated and from this the total cost of management for each patient has been calculated.

Received 6 December 1991; and in revised form 12 October 1992.

### Patients and methods

### *Patients*

A computer database containing information on all patients with breast cancer who have attended the Clinical Oncology Unit at Guy's Hospital was used to identify patients who were known to have died during the last 3 months of 1988 ($n = 25$) or at any time during 1990 ($n = 88$). The medical records of all 25 patients who had died in the first period and a random selection of records of 50 patients from the second period were examined for information regarding management of advanced breast cancer. Twenty-five patients were excluded from further analysis, 13 of whom died of other causes without ever having evidence of relapse of breast cancer. Four patients received treatment for primary breast cancer at Guy's, but treatment for advanced disease was given exclusively at other hospitals. In the remaining eight cases, care for advanced disease was shared electively with other hospitals, as the patients lived outside the local health district. Accurate information on resource usage was not available for these patients. Hence, 50 patients whose management for advanced cancer was undertaken principally at Guy's Hospital form the basis of this study. These included patients who were managed in conjunction with a hospice or home care support team and patients who presented as emergencies to other hospitals and were then referred to Guy's for subsequent management.

Detailed information was retrieved from the case records of each of the 50 patients in the study for the period between the development of advanced disease and death. For patients who had initially presented with operable disease, the date of diagnosis of distant metastasis or inoperable locoregional recurrence was taken as the onset of advanced disease. Contralateral breast tumours and operable recurrences within a conserved breast diagnosed before this time were not considered as advanced disease. For patients who presented with inoperable locally advanced disease and for those with overt metastases at the time of diagnosis, the date of presentation to the unit was taken as the date of advanced disease.

The following information was recorded for each patient: Duration of in-patient admissions, together with the principal indication for each admission; Number of out-patient visits to surgical, radiotherapy and medical oncology clinics; All surgical, radiotherapy, endocrine and chemotherapy treatments administered; All laboratory and radiological investigations; Participation in research studies; Place of death; Cause of death and whether a hospice or home care team was involved in the patient's management. Emergency management in other hospitals was noted, but excluded from the analysis of total costs.

### Costing methodology

*In-patient costs*

Patients were managed on a ward designated for patients with breast diseases (The Hedley Atkins Unit). The cost of an overnight stay was estimated by calculating the total cost of running the ward and dividing this by the total number of overnight stays for the year 1988/89. No account was taken of the complexity of care on the ward for an individual patient. In a small minority of cases (5% - see Results) patients were managed on other specialist wards (orthopaedic or neurosurgical). For simplicity, in-patient costs have been taken to be the same as those for the oncology ward. Salaries including employers National Insurance Contribution and London weighting for staff employed on the ward (e.g. nurses) were calculated at a March 1991 pay and price base. The proportion of the working week attributable to the management of Breast Unit in-patients was calculated for other staff (e.g. doctors, physiotherapists, dieticians) and costs were calculated accordingly. This included staff employed by the University of London or funded by the Imperial Cancer Research Fund. In addition, both direct (e.g. portering and domestic services) and indirect overheads (e.g. hospital administration) were included in the calculation of costs. A further 19% was added to the calculated cost to cover the capital charges applicable to the Guy's and Lewisham NHS Trust in the year 1991-92.

*Medical and surgical out-patient costs*

Each member of staff involved in out-patient clinics was asked to identify the amount of time devoted to that clinic, including time spent on supporting administration. A total cost for each clinic including consumables (other than cytotoxic drugs), direct and indirect overheads and capital charges was calculated and divided by the average number of patients attending that clinic to give a cost per patient. Separate costs were established for medical clinics relating to endocrine treatment and those in which chemotherapy was administered.

*Radiotherapy costs*

Radiotherapy treatment and out-patient clinic visits are under the management of the South East London Radiotherapy Centre (covering Guy's, St Thomas' and King's College Hospitals), whereas in-patient costs for radiotherapy at Guy's Hospital fall within the budget of the Oncology Directorate. In this study, radiotherapy treatment and out-patient clinics were not costed separately. The cost of an out-patient visit was arbitrarily taken to be the same as a visit to the medical endocrine treatment clinic. The cost of a fraction of radiotherapy (£37) used in the current analysis was based on the findings of Goddard and coworkers in a recent study of the cost of palliative radiotherapy at Mount Vernon Hospital, Northwood, Middlesex (Goddard *et al.*, 1991). This figure is in line with the £30 cost calculated in 1988 by Goddard and Hutton based on the reports of Greene (1983) and Atherton (1984).

*Chemotherapy*

The number of cycles of each chemotherapeutic regimen administered and the duration in months of each endocrine treatment was established for each patient. Current (1991) costs of drugs at Guy's Hospital were used for the calculation of total costs of chemotherapy and endocrine therapy.

*Surgery*

The average length of each type of operation performed over a 6 month period was determined by using the Theatre Financial Information Project (FIP) system which records the details of each operation performed in the Guy's Theatres. The average length of each operation multiplied by the staff cost per hour gives the staff cost per operation. To this were added the costs of anaesthetic consumables and surgical and medical equipment used together with a proportion of the direct and indirect overheads for running the operating theatre. Capital charges (19%) were added to this cost.

*Radiology and laboratory investigations*

All investigations undertaken on each patient were recorded. Costings for each item were kindly provided by the Pathology and Radiology Directorates in order to calculate the total cost of investigations for each patient. These include the cost of consumables, staff and direct overheads.

*Sensitivity analysis*

Cost estimates are subject to error. The impact of changes on the costs of individual items was therefore assessed by performing a sensitivity analysis. The sensitivity to variations in costs was defined as the percentage change that would be required to increase overall cost by 1%.

### Results

Thirty-one of the patients had initially presented with operable breast cancer, 12 presented with locally advanced disease and seven had overt metastases at the time of first diagnosis. Nine patients lived in the health district served by Guy's, 33 in other districts within the South East Thames Region and eight in other health regions. Mean age at first diagnosis was 58 years (range 32 to 85) and at the onset of advanced disease was 60.5 years (range 32 to 87). Median duration of advanced disease was 17 months (range 7 days to 12 years). Twenty-three of the patients died at Guy's, 14 at home and 13 in a hospice.

*In-patient stays*

The 50 patients spent a total of 1,677 nights in hospital, 1,588 of which (95%) were spent on the designated oncology ward. The mean number of overnight stays in hospital was 34 (range 0-133: Table I). In most (>80%) cases it was possible to identify a principal indication for each admission from the in-patient records and discharge summary. Bone metastases causing pain, immobility, pathological fractures, hypercalcaemia or spinal cord compression accounted for 36% of all overnight stays. In many cases patients received

**Table I** In-patient stays

| *Indication* | *No. of patients* | *Nights in hospital Total* | *%* |
|---|---|---|---|
| Assessment/staging[a] | 24 | 134 | 8 |
| Chemotherapy/neutropenia | 19 | 76 | 5 |
| Bone/spinal cord compression[b] | 12 | 602 | 36 |
| Lung/pleural/pericardial complications | 11 | 239 | 14 |
| Liver metastases | 6 | 45 | 3 |
| Brain metastases | 6 | 220 | 13 |
| Metastases at multiple sites | 22 | 274 | 16 |
| Other surgery[c] | 6 | 65 | 4 |
| Research | 4 | 42 | 3 |
| Total | | 1677 | |

[a]Includes diagnostic surgical procedures (e.g. excision biopsy of a lymph node). [b]Includes orthopaedic surgery. [c]e.g. palliative mastectomy, but excluding orthopaedic/neurosurgical procedures.

palliative radiotherapy during these admissions, but were not sufficiently mobile to attend the radiotherapy department as out-patients. In particular, eight patients spent a total of 561 nights (range 25–120) in hospital because of spinal cord compression or pathological fractures.

Illness related to cerebral, liver, pulmonary, pleural or pericardial metastases or a combination of these sites of disease accounted for 46% of time spent as in-patients. In some instances patients received chemotherapy during the course of an admission, but the treatment did not add to the duration of stay.

Twenty-seven of the 50 patients received cytotoxic chemotherapy for advanced disease. In general this was given as an out-patient except for the first cycle when overnight admission was recommended for acute side effects to be observed. Administration of chemotherapy and admissions resulting from chemotherapy-induced myelosuppression accounted for 76 of the 1,677 (5%) nights in hospital.

All patients who had participated in clinical research trials were identified. When this was the reason for an admission, or when an admission was prolonged for research purposes (e.g. to collect blood samples for pharmacokinetic studies), the extra time in hospital was calculated. A total of 42 nights in hospital were attributable to research, 37 of these in one patient who received two experimental drug treatments.

*Specific treatment*

Twelve patients underwent some form of palliative surgery (e.g. palliative mastectomy, talc pleurodesis or orthopaedic procedures) during the course of their illness. Thirty-three patients received a total of 75 courses of radiotherapy (765 fractions). Forty-five received some form of systemic therapy (18 endocrine treatment only; five chemotherapy only and 22 both chemotherapy and endocrine therapy). The mean duration of endocrine therapy for the 38 patients treated was 16 months (range 1 to 96). The 27 patients treated with cytotoxic agents received a total of 225 cycles (range 1–43). The total number of clinic visits were as follows: surgical 170; radiotherapy 354; endocrine 624 and chemotherapy 301.

*Investigations*

Details of the number of different radiological and laboratory investigations undertaken on the 50 patients are shown in Table II and III. Radionuclide bone scans accounted for 41% of the total cost of radiological investigations and bone radiographs a further 20%. Biochemical tests accounted for 44% of the cost of laboratory investigations.

**Table II** Radiological investigations

| Investigations | Total number | Mean per patient | % of total cost of radiology |
|---|---|---|---|
| Bone radiograph | 420 | 8.4 | 20 |
| Chest radiograph | 299 | 6.0 | 10 |
| Radionuclide bone scan | 144 | 2.9 | 41 |
| Radionuclide liver scan | 34 | 0.7 | 8 |
| Ultrasound | 30 | 0.6 | 3 |
| CT scan | 16 | 0.3 | 7 |
| Gated cardiac scan | 7 | 0.1 | 3 |
| Other[a] | – | – | 8 |
| | | Total | 100% |

[a]Includes: other radiographs, mammography, myelography, magnetic resonance imaging, phlebography and echocardiography.

**Table III** Laboratory investigations

| Investigations | Total number | Mean per patient | % of total cost of laboratory costs |
|---|---|---|---|
| Haematology | 828 | 16.6 | 28 |
| Biochemistry | 729 | 14.6 | 44 |
| Microbiology | 134 | 2.7 | 10 |
| Histology/cytology | 64 | 1.3 | 18 |
| | | Total | 100% |

*Costs*

The cost of an overnight stay was calculated to be £129 (inclusive of overheads and capital charges). Those for visits to the surgical, endocrine and chemotherapy clinics were £12, £23 and £68 respectively. For the reasons given in the Methods section, visits to radiotherapy clinics were arbitrarily set to be the same as for a visit to the endocrine treatment clinic. The cost of each fraction of radiotherapy was taken to be £37 (see Methods).

Overall costs of treatment for the 50 patients are shown in Table IV, together with the proportion of costs attributable to separate aspects of management. The mean cost per patient was £7,620. The range of costs was wide (£317 to £27,860). The most expensive management was that of a patient who spent 133 days in hospital, attended clinics on 59 occasions, received 18 cycles of chemotherapy and three courses of palliative radiotherapy (26 fractions) over a period of 57 months.

*Sensitivity analysis*

Sensitivity analysis demonstrated that an increase of 2% in the cost of in-patient stays (either because of higher prices or because of increased use of resources) would increase the total cost by 1%. The same overall change would be incurred by an increase of 9% in out-patient clinic costs, by an increase of 12% in cytotoxic drug costs, by an increase of 12% in the cost of radiological investigations or by a 30% increase in laboratory investigation costs. A 60% change in the cost of endocrine drugs would be required to change the total cost by 1%.

### Discussion

In this study an attempt has been made to calculate the total usage of hospital resources and the overall cost of care for patients with advanced breast cancer managed within a specialised NHS unit. Resource usage was calculated by abstracting detailed information from case records maintained specifically by the Breast Unit and Radiotherapy Department. As has been noted by others (Hurley et al., 1992), it is impossible to guarantee that these records were complete. However, significant underrecording of resource usage is considered unlikely.

Previous economic studies of patients with advanced breast cancer have generally focussed either on the use of hospital beds (Mattsson et al., 1979) or on the cost of out-patient cytotoxic drug administration (Friedlander & Tattersall, 1982; Rees, 1985). Two studies, one from Australia, the other from The Netherlands, have, however, recently been published in which total resource usage has been calculated (Hurley et al., 1992; de Koning et al., 1992). The data presented by de Koning and coworkers for 68 patients with advanced breast cancer who died between 1985 and 1989 can

**Table IV** Costs of treatment for all 50 patients

| | Total (£) | Mean cost per patient | % |
|---|---|---|---|
| In-patient stays | 216,333 | 4,327 | 57 |
| Out-patient clinics | 43,714 | 874 | 12 |
| Surgical treatment | 9,000 | 180 | 2 |
| Endocrine drugs | 6,434 | 129 | 2 |
| Chemotherapy drugs | 31,964 | 639 | 8 |
| Radiotherapy | 28,305 | 566 | 7 |
| Radiological investigations | 32,155 | 643 | 9 |
| Laboratory investigations | 13,105 | 262 | 3 |
| Total | 381,010 | 7,620 | 100 |

be directly compared with the findings in the current study (Table V). Mean duration of stay in hospital was somewhat longer in The Netherlands (45 days) than at Guy's (34 days). In other respects, use of resources appears remarkably similar.

Costing estimates involve assumptions and are always subject to error and possible criticism in studies of this type (Hurley *et al.*, 1992). In some countries, standard tariffs are available and are used for costing studies (de Koning *et al.*, 1992). In the UK such information is not available (Rees, 1985). The approach adopted in the current study has therefore been to calculate costs for individual components of care wherever possible. Where this was not possible, as in the case of radiotherapy costings, the best available published data for the UK was used (Goddard *et al.*, 1991). Because of the likelihood of errors involved in the estimation of costs, a sensitivity analysis was undertaken. Apart from the cost of in-patient stays, moderate errors in the costs of separate components of care make only small changes to the overall costs.

The overall mean cost of hospital care calculated in this study (£7,620) can be compared with results from other countries. In the United States, the cost of treatment in the last 6 months of life for breast cancer patients covered by the Medicare programme was estimated to be $15,137 at 1984 prices (Baker *et al.*, 1991). In the study from Australia (Hurley *et al.*, 1992), the median cost for 89 patients who had died with metastatic breast cancer was Aus $11,948 (1988). The authors noted that because of skewing of costs, mean costs are higher than median costs. In that study hospital visits comprised 54% of total costs and investigations 24%.

Costs in the current study can most closely be compared with those reported from The Netherlands (de Koning *et al.*, 1992). Excluding the costs of Nursing home stays (which were not assessed for our patients), de Koning *et al.* reported a mean total cost of hospital care of US $15,850 (approximately £8,450). Importantly, the proportions of total costs attributable to different areas were very closely matched. Thus, in-patient stays and clinic visits (including specialist fees) accounted for 70% of total cost at Guy's and 71% in The Netherlands. Diagnostic procedures accounted for 12% and 11% of total costs respectively.

How does the estimated cost for caring for patients with advanced breast cancer compare with the cost of treating other conditions within the NHS? Lobo and coworkers (1991) found that the cost of induction treatment for acute myelogenous leukaemia was between £15,283 and £18,740. In a study of surgical workload and cost, Ellis (1991) calculated that the in-patient treatment costs (including surgery) for transurethral prostatectomy and cholecystectomy were each in the region of £1,200.

The largest component of the total cost of managing advanced breast cancer is attributable to time spent as an in-patient in hospital, staff salaries accounting for most of this cost. The findings of this study demonstrate that most of the time spent as an in-patient is due to complications arising from the disease itself rather than to its treatment. Bone metastasis, the commonest site of spread in patients with breast cancer, can lead to prolonged hospitalisation, particularly when accompanied by spinal cord compression. Cerebral metastases, a less common site of spread, accounted for a disproportionate amount of time in hospital.

As this study was conducted in the setting of a research oriented tertiary referral unit, it was considered important to estimate the extra time spent in hospital for clinical research. Teaching hospitals receive supplementary funding to cover some of the expenses associated with clinical research through the Service Increment for Teaching and Research (SIFTR). In the past, the extra costs associated with clinical research have usually been poorly defined. In the future, however, it is likely that both the NHS and the charitable bodies funding clinical research will require much closer scrutiny of the costs of such clinical research. In the current study, the proportion of in-patient stays related to research was 3%, 37 of the 42 days resulting from experimental treatment given to one patient. Clearly, major differences in the proportion of in-patient time attributable to research could result from implementation of new clinical trials.

A widely held belief about cancer treatment is that it is expensive because of the high cost of cytotoxic drugs. Cytotoxic agents are certainly more expensive than many other drugs and the costs are easy to identify, but as can be seen from this study, they account for a relatively small proportion (8%) of the total cost of patient management. Palliative radiotherapy accounted for a similar proportion of the total cost (7%). The cost of cytotoxic drugs combined with the cost of running the chemotherapy clinic and the cost of time spent in hospital related to chemotherapy accounted for 16% of the total cost. In this study, it was not possible to assess the relative benefits of the different treatments. It is to be hoped that such assessments will become possible when the results of prospective audit studies are available (Rubens, 1991).

What are the practical implications of a study such as this? Clearly any measures that can reduce time spent in hospital are likely to result in major savings but only if the beds are closed or are put to alternative use. In this unit, investigations are carried out on an out-patient basis whenever possible although for some patients who are unwell and have long distances to travel this is impractical. Similarly, radiotherapy is given as an out-patient whenever possible, but for patients who are sick or immobile, this is not feasible or humane. No account was taken in this study of the costs of patient transport. One way in which in-patient stays can potentially be reduced is by adopting shorter schedules of radiotherapy, involving fewer fractions of treatment. The equivalence of the benefit in terms of palliation of shorter courses of radiotherapy is now becoming apparent (Borgelt *et al.*, 1980; Gelber *et al.*, 1981), and has led to changes in treatment policy within the unit. It should be noted that the patients in this study first developed advanced breast cancer between 1978 and 1990.

Prevention and early detection of the complications of bone metastases would have a major impact on the overall cost of treatment. Studies are currently in progress to assess the role of bisphosphonates in this regard. If patients who are at high risk of developing bone metastases can be identified and if these agents can reduce the incidence of pathological fractures, this could lead both to improvement in quality of life for breast cancer patients and to considerable savings for the NHS. Early detection of spinal cord compression should also be a priority area for clinical research. If patients could be identified early, perhaps by the use of magnetic resonance imaging, the costs of an expensive investigation might of offset by a reduction in the time spent in hospital.

Finally, close collaboration with hospices and home care support teams can also help to reduce costs and, we believe,

**Table V** Comparison of resource usage in two different studies

| | *Guys* (n = *50*) | *Netherlands* (n = *68*) |
|---|---|---|
| Mean survival | | |
| months | 27 months | 21.4 |
| Median survival | | |
| months | 17 months | 20.3 |
| Proportion of patients receiving: | | |
| Radiotherapy | 66% | 59% |
| Endocrine therapy | 80% | 84% |
| Chemotherapy | 54% | 69% |
| Mean duration of endocrine therapy | 12 months | 14 months |
| Mean duration of chemotherapy | 3.5 months | 4 months |
| Mean number of days in hospital | 34 | 45 |
| Mean number of diagnostic procedures: | | |
| X-rays | 14.4 | 22 |
| Isotope scans | 3.7 | 2 |
| CT scans | 0.3 | 0.8 |
| Pathology procedures | 1.3 | 1.9 |

significantly improve quality of life for patients and their relatives. The benefit to the patients and to the NHS of such services, many of which are funded from charitable sources, is considerable.

We are grateful to Professor M.N. Maisey and Mr N. Bosanquet for providing information on the costs of radiological procedures and to Professor D.A. Levison and Mr P. Wozencroft for information on laboratory costs. Ms C. Ashley and Mr R. White, from the Finance Department at Guy's Hospital, kindly provided information relating to the cost of indirect overheads and capital charges.

### References

ATHERTON, L. (1984). The cost of radiotherapy treatments on a linear accelerator (letter). *Br. J. Radiol.*, **57**, 106-107.

BAKER, M.S., KESSLER, L.G., URBAN, N. & SMUCKER, R.C. (1991). Estimating the treatment costs of breast and lung cancer. *Med. Care*, **29**, 40-49.

BORGELT, B., GELBER, R., KRAMER, S., BRADY, L.W., CHANG, C.H., DAVIS, L.W., PEREZ, C.A. & HENDRICKSON, F.R. (1980). The palliation of brain metastasis: final results of the first two studies by the radiation therapy oncology group. *Int. J. Radioth. Oncol. Biol. Phys.*, **6**, 1-9.

DE KONING, H.J., VAN INEVELD, B.M., DE HAES, J.C.J.M., VAN OORT-MARSSEN, G.J., KLIJN, J.G.M. & VAN DER MAAS, P.J. (1992). Advanced breast cancer and its prevention by screening. *Br. J Cancer*, **65**, 950-955.

ELLIS, B.W. (1991). Management important of common treatments: contribution of top 20 procedures to surgical workload and cost. *Br. Med. J.*, **302**, 882-884.

FRIEDLANDER, M.L. & TATTERSALL, M.H.N. (1982). Counting the costs of cancer therapy. *Eur. J. Clin. Oncol.*, **18**, 1237-1241.

GELBER, R.D., LARSON, M., BORGELT, B.B. & KRAMER, S. (1981). Equivalence of radiation schedules for the palliative treatment of brain metastases in patients with favourable prognosis. *Cancer*, **48**, 1749-1753.

GODDARD, M. & HUTTON, J. (1988). *The Costs of Radiotherapy in Cancer Treatment*. Discussion Paper 48: University of York Centre for Health Economics.

GODDARD, M., MAHER, E.J., HUTTON, J. & SHAH, D. (1991). Palliative radiotherapy – counting the costs of changing practice. *Health Policy*, **17**, 243-256.

GREENE, D. (1983). The cost of radiotherapy treatments on a linear accelerator. *Br. J. Radiol.*, **56**, 189-191.

HURLEY, S.F., HUGGINS, R.M., SYNDER, R.D. & BISHOP, J.F. (1992). The cost of breast cancer recurrences. *Br. J. Cancer*, **65**, 449-455.

LOBO, P.J., POWLES, R.L., HANRAHAN, A. & REYNOLDS, D.R. (1991). Acute myeloblastic leukaemia – a model for assessing value for money for new treatment programmes. *Br. Med. J.*, **302**, 323-326.

MATTSSON, W., GYNNING, I., CARLSSON, B. & MAURITZON, S.-E. (1979). Cancer chemotherapy in advanced malignant disease. *Acta Radiol.*, **18**, 509-520.

REES, G.J.G. (1985). Cost effectiveness in oncology. *Lancet*, **ii**, 1405-1408.

RUBENS, R.D. (1991). Auditing palliative cancer chemotherapy. *Eur. J. Cancer*, **26**, 1023-1025.